# Hashtag bone: detrimental effects on bone contrast with metabolic benefits one and five years after Roux-en-Y gastric bypass

**DOI:** 10.1590/1414-431X2021e11499

**Published:** 2021-12-03

**Authors:** M.A.V.S.D. Alencar, I.M. de Araújo, L.T. Parreiras-e-Silva, M.H. Nogueira-Barbosa, W. Salgado, J. Elias, C.E.G. Salmon, F.J.A. de Paula

**Affiliations:** 1 Departamento de Medicina Interna, Faculdade de Medicina de Ribeirão Preto, Universidade de São Paulo, Ribeirão Preto, SP, Brasil; 2 Departamento de Imagens Médicas, Hematologia e Oncologia Clínica, Faculdade de Medicina de Ribeirão Preto, Universidade de São Paulo, Ribeirão Preto, SP, Brasil; 3 Departamento de Cirurgia e Anatomia, Faculdade de Medicina de Ribeirão Preto, Universidade de São Paulo, Ribeirão Preto, SP, Brasil; 4 Departamento de Física, Faculdade de Filosofia, Ciências e Letras de Ribeirão Preto, Universidade de São Paulo, Ribeirão Preto, SP, Brasil

**Keywords:** Osteoporosis, Bone mineral density, Insulin resistance, Visceral adipose tissue, Subcutaneous adipose tissue, Magnetic resonance imaging

## Abstract

Bone loss is a potential adverse consequence of rapid and sustained weight loss after bariatric surgery. The aim of the present study was to evaluate the bone mass, body fat distribution, and metabolic parameters in women submitted to Roux-en-Y gastric bypass (RYGB). The study included the following three groups: one group of lean women (control [C] group) and two groups of obese women, one evaluated one year (B1) and the other five years (B5) after RYGB. Dual-energy X-ray absorptiometry and magnetic resonance imaging were used to determine bone mineral density (BMD; lumbar spine, total hip, and femoral neck) and abdominal fat content (subcutaneous [SAT] and visceral [VAT] adipose tissues, and intrahepatic lipids [IHL]). The BMD/body mass index ratio was lower in the B5 compared with the C group at all sites. Serum C-terminal telopeptide of type I collagen (CTX) levels were higher in the B1 and B5 groups compared with the C group. Individuals submitted to RYGB showed greater SAT but similar VAT and IHL values compared with those in the C group. However, the B5 group had higher mean parathyroid hormone levels compared with the other two groups. Individuals submitted to RYGB presented increased levels of CTX and low BMD for body weight than those in the C group, suggesting that bone catabolism is a persistent alteration associated with RYGB. In conclusion, the long-lasting metabolic benefits obtained with RYGB in obesity are counterbalanced by a persistent catabolic effect of the procedure on bone and mineral metabolism.

## Introduction

Obesity is a chronic noncommunicable disease with a unique social disseminating process (1). Obesity is classified as a global epidemic disorder by the World Health Organization (2), and is a risk factor for metabolic, cardiovascular, and proliferative diseases (3). Bariatric surgery is the most effective treatment for obesity, leading to weight loss in a considerable proportion of patients and improving metabolic abnormalities even before substantial changes in body weight (4). Evidence suggests that Roux-en-Y gastric bypass (RYGB) changes the profile of hormones secreted by the gastrointestinal system, accelerating metabolic improvement even before weight loss (5). Currently, approximately 200,000 bariatric surgeries are performed annually in the United States (6) and 100,000 in Brazil (7). According to clinical surveys, short term mortality is lower when these surgeries are performed at a specialized hospital (2). In the long term (∼7 years), the number of deaths is 40% lower in obese patients submitted to bariatric surgery compared with obese controls (8,9). These rates are far higher than those achieved by individuals with obesity treated with noninvasive therapies, which also demand great and persistent effort from patients. These observations justify the increasing number of bariatric surgeries performed in several countries (10), although this is an outdated form of therapy for functional diseases. The improvement in life expectancy in patients submitted to bariatric procedures allows the evaluation of adverse consequences associated with these procedures, including their effects on bone.

Body weight correlates positively with bone mass. Muscle and adipose tissue contribute to bone remodeling through mechanical and endocrine modulation (11,12). The impact of adipokines (e.g., leptin and adiponectin) on the skeleton is complex and has not been fully elucidated. For instance, leptin appears to have opposing actions on bone by directly stimulating osteoblasts and indirectly inhibiting bone formation via central activation of the sympathetic system (13). In 2010, the association between fractures and obesity gained importance with the finding that obesity has no protective effect on the occurrence of fractures (14). The relationship between bone and adipose tissue is complex, and several factors are implicated in fracture risk in obesity. The mechanical action of increased fat mass has a positive effect on bone, and the increased estrogen levels associated with obesity favor the maintenance of bone mass. In contrast, the chronic inflammatory status and the endocrine profile of adipocytes in obesity can have a detrimental effect on bone, as previously described (15). Moreover, bariatric surgery has great beneficial effects on insulin resistance but negative effects on the skeleton, increasing bone resorption markers (16) and leading to bone loss (17), changes in bone histomorphometry (18), and increased occurrence of fractures (19). Despite this, measures to mitigate bone weakness are lacking (20).

Weight loss is the most efficient approach to prevent type 2 diabetes mellitus (T2DM) (21). Insulin resistance and its related disorder, nonalcoholic fatty liver disease (NAFLD), are two of the most common and severe complications associated with obesity. According to previous studies, hepatic accumulation of fat is negatively associated with bone mass in individuals with short bowel syndrome (22,23). However, studies evaluating the association of NAFLD and bone in individuals with obesity and T2DM have shown conflicting results, suggesting that factors other than the amount of intrahepatic lipids (IHL) mediate the relationship between liver and bone (24,25).

Few data are available in the literature regarding long-term quantitative evaluation of adipose tissue after RYGB and the relationship of adipose tissue with the skeleton in the short- and long-term. Based on these considerations, the present cross-sectional study was designed to evaluate the bone mineral density (BMD), body composition, and abdominal adiposity in two groups of individuals submitted to bariatric surgery, the first at one year and the other at five years after the procedure. Abdominal fat quantification was performed from measurements of IHL, visceral adipose tissue (VAT), and subcutaneous adipose tissue (SAT). The results obtained in these two groups were compared with those of individuals with normal weight and no metabolic disease. An additional aim of the study was to evaluate the relationship of BMD and IHL levels with serum adiponectin and leptin levels.

## Material and Methods

### Subjects

The protocol of the study was approved by the institutional review board of the University Hospital of Ribeirão Preto Medical School (FMRP-USP; protocol number 054941/2014, CAAE 32971214.9.0000.5440). All subjects signed a written informed consent to participate in the study after receiving information about the risks and discomforts involved with the procedures. Due to the characteristics of the study, we adopted a convenience sampling model. The study comprised 54 premenopausal or postmenopausal women allocated to three groups: a) control (C), n=21; b) obese, one year after RYGB (B1), n=16; and c) obese, five years after RYGB (B5), n=17. The groups were matched by age and height. The mean values of body weight and BMI were similar between the B1 and B5 groups but were significantly higher in both of these groups compared with group C (P<0.05). Menopausal status was determined by clinical parameters. Individuals in the C group were people from the community who spontaneously accepted the invitation to participate in the study and were subsequently matched for height and age with the patients in the bariatric groups. Subjects from the B1 and B5 groups were recruited from the Bariatric Surgery Outpatient Clinic of the University Hospital (HC-FMRP-USP).

The inclusion criteria were female sex and age 20-70 years. Specifically, participants in the bariatric surgery groups had undergone the procedure one year (B1) or five years (B5) before enrollment. The exclusion criteria in all groups were the presence of chronic diseases known to affect bone metabolism, pregnancy, abnormal thyroid function, hypothalamic or pituitary disorders, use of glucocorticoids or osteoporosis medications (bisphosphonates, denosumab, teriparatide, strontium ranelate, or calcitonin), early menopause, smoking, or alcoholism.

### Laboratory analysis

Blood samples were obtained between 8 and 9 a.m. after a 12-h overnight fast. Measurement of serum levels of total glucose, albumin, inorganic phosphorus, alkaline phosphatase, creatinine, aspartate aminotransferase (AST), alanine aminotransferase (ALT), and calcium was performed using an automatic biochemical analyzer (CT 600i, Wiener Lab Group, Argentina). Serum levels of 25-hydroxyvitamin D (25-OHD; Liaison, DiaSorin, Italy), intact parathyroid hormone (PTH; Immulite 2000, Siemens, USA), and insulin-like growth factor 1 (IGF1; Immulite 2000, Siemens) were determined by chemiluminescence. Levels of C-terminal telopeptide of type I collagen (CTX) were determined by electrochemiluminescence (Cobas E 411, Roche Diagnostics, USA). Serum osteocalcin (hOST-EASIA Diasource, Belgium), leptin (Quidel, TECO Medical Group, Switzerland), and adiponectin (Millipore, USA) were determined by enzyme immunoassay. All intra-assay and inter-assay coefficients of variation were lower than 10 and 20%, respectively. Insulin resistance was estimated using the homeostasis model assessment of insulin resistance (HOMA-IR) according to the following formula: fasting serum insulin (μIU/mL) × fasting plasma glucose (mM) / 22.5 (26).

### Dual-energy X-ray absorptiometry

Dual-energy X-ray absorptiometry (Hologic Discovery Wi, QDR series, USA) was used to determine BMD in the lumbar spine (L1-L4), total hip, and femoral neck, with values reported in g/cm^2^. BMD Z-scores were determined to categorize bone mass into adequate (>-2.0) or inadequate (≤-2.0) for age. Precision errors for L1-L4, femoral neck, and total hip BMD were 0.9, 1.8, and 1.46%, respectively. We also analyzed the BMD corrected for body mass index (BMD/BMI ratio) to assess whether the amount of bone loss was an adjustment for the body weight loss.

### Abdominal magnetic resonance imaging

A phased-array torso coil was used to acquire abdominal images in a 1.5-T magnetic resonance scanner (Philips Achieva, Philips Medical Systems, The Netherlands). A coronal turbo-spin-echo T2-weighted breath-hold sequence was applied to localize the subsequent scan volumes. Two sets of axial gradient double-echo T1-weighted breath-hold sequences were acquired consecutively in-phase (echo time=4.2 ms) and out-of-phase (echo time=2.1 ms, slice thickness=6.0 mm), one including the upper abdomen and another centered on the umbilical region.

In order to calculate the IHL value from the average signal intensity (SI) in each region of interest (ROI) and the pair of in-/out-of-phase images, the following formula was used: IHL=(in-phase SI − out-of-phase SI) / (2 x in-phase SI). Manual segmentation of the liver at the level of the main portal vein was performed to select four ROIs as representative segments. Visceral and subcutaneous adipose tissue areas (mm^2^) were defined using a semiautomatic segmentation of an axial slice at the level of the umbilicus using the software Display (http://www.bic.mni.mcgill.ca/software/Display/Display.html), as previously detailed (22,24).

### Statistical analysis

Data in all three groups were analyzed using a simple variance test (one-way ANOVA), followed by the Duncan post-test, using the general linear model procedures (PROC GLM) of SAS, version 9.4 (SAS Institute Inc., USA).

## Results

Table 1 shows the clinical characteristics (age, height, BMI, and biochemical assessment) of the participants in the three groups. The percentages of postmenopausal women in each group were 42% in the C group, 43% in the B1 group, and 29% in the B5 group. The means±SD weight loss and the rate of weight loss in the bariatric groups were, respectively, 41.4±8.3 kg and 33.4±6.4% in the B1 group and 43.5±17.5 kg and 32.9±9.3% in the B5 group. The mean BMI decreased by 15.8±3.2 kg/m^2^ (from 47.1 to 31.3 kg/m^2^) in the B1 group and by 17.3±7.1 kg/m^2^ (from 50.5 to 33.2 kg/m^2^) in the B5 group. The three groups had similar serum levels of glucose, ALT, and AST. Although the serum levels of corrected calcium were also similar in all three groups, the serum 25-OHD levels were higher in the B1 compared with the C and B5 groups, while serum levels of PTH were higher in the B5 compared with the C and B1 groups (P<0.05). Serum glucose levels ranged from 73 to 96 mg/dL in the C group, from 70 to 91 mg/dL in the B1 group, and from 71 to 115 mg/dL in the B5 group. Serum IGF1 levels were lower in the B5 compared with the C group (P<0.05) but were similar between the B1 and B5 groups. No differences were observed regarding mean serum levels of insulin, adiponectin, or leptin across groups.

**Table 1:** Clinical characteristics of the control group, and obese women evaluated one year (B1) and five (B5) years after Roux-en-Y gastric bypass.

	Control group (n=21)	B1 group (n=16)	B5 group (n=17)
Age (years)	43.3±13.9	42.4±12.5	43.9±7.9
Height (m)	1.64±0.09	1.63±0.08	1.61±0.06
Body mass index (BMI) (kg/m^2^)	23.0±2.3^a^	31.3±5.8	33.2±6.5
Glucose (mg/dL)	87±6	83±6	86±10
Insulin (mIU/mL)	6.9±2.9	6.2±1.9	8.0±6.6
HOMA-IR	1.5±0.6	1.3±0.4	1.6±1.6
Creatinin (mg/dL)	0.8±0.1^b^	0.8±0.1	0.7±0.1
Albumin (mg/dL)	4.4±0.2^b^	4.2±0.2	4.1±0.3
Corrected calcium (mg/dL)	9.6±0.4	9.5±0.4	9.6±0.6
Phosphorus (mg/dL)	3.4±0.4	3.7±0.4	3.6±0.4
PTH (pg/mL)	35.3±16.0	42.4±22.1	61.0±26.1^c^
25-OHD (ng/mL)	25.2±10.4	36.7±17.4^d^	24.7±6.1
Alkaline phosphatase (U/L)	174±47	197±46	204±53
AST (U/L)	20±4	24±8	20±6
ALT (U/L)	17±8	23±10	16±8
IGF1 (ng/mL)	197±92^b^	155±70	105±42
Osteocalcin (OC) (ng/mL)	12.8±7	14.5±5	12.4±7
CTX (ng/mL)	0.41±0.16^a^	0.81±0.25^d^	0.62±0.18
Leptin (ng/mL)	33.6±24.3	25.8±16.5	29.7±15.7
Adiponectin (ng/mL)	16.9±14.2	22.3±14.8	26.5±23.0

Data are reported as means±SD. ^a^P<0.05 compared to B1 and B5, ^b^P<0.05 compared to B5, ^c^P<0.05 compared to Control and B1, ^d^P<0.05 compared to Control and B5 (ANOVA). PTH: parathyroid hormone, AST: aspartate aminotransferase, ALT: alanine aminotransferase, 25-OHD: 25-hydroxyvitamin D, IGF1: insulin-like growth factor 1, CTX: C-terminal telopeptide of type I collagen.

**Figure 1:**
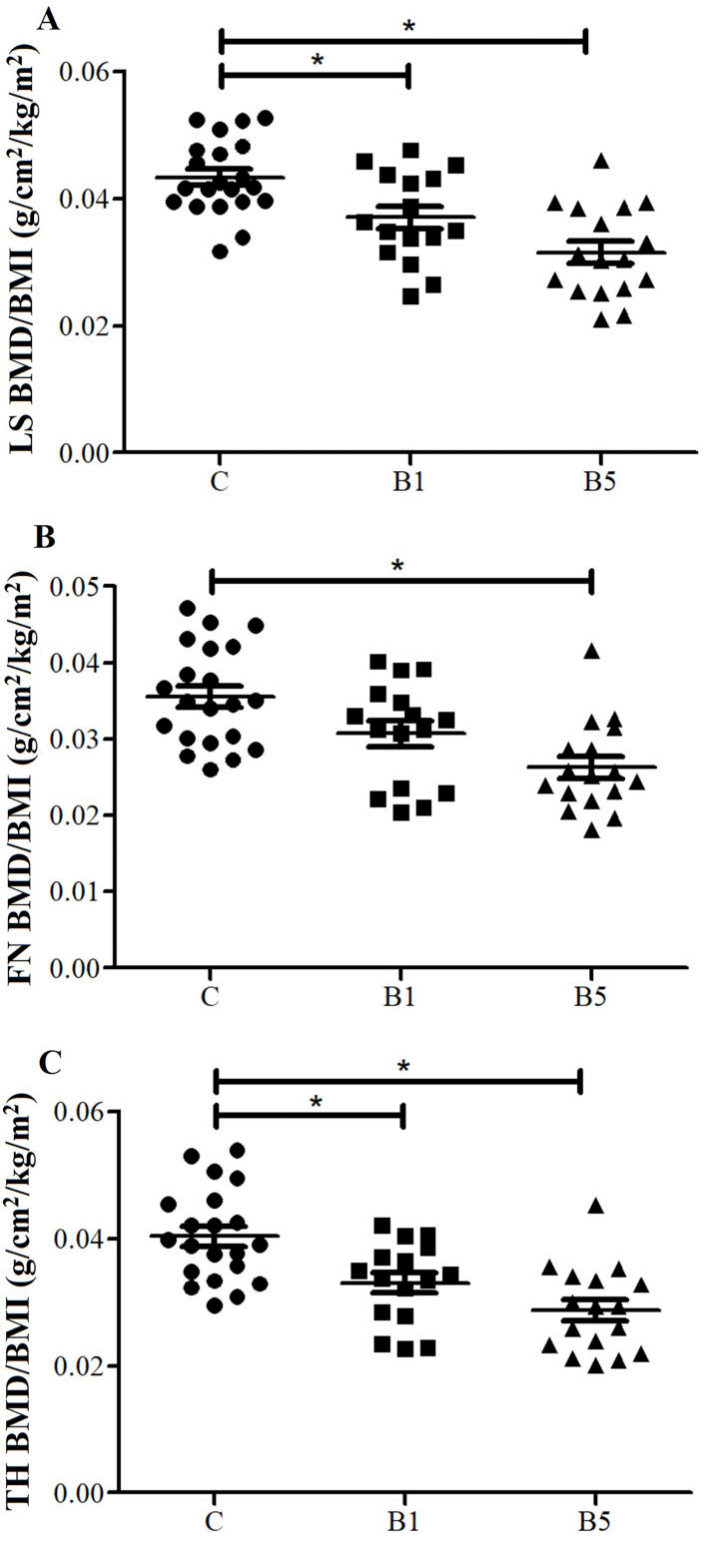
Distribution of (**A**) lumbar spine bone mineral density/body mass index (LS BMD/BMI) ratio, (**B**) femoral neck bone mineral density/body mass index ratio (FN BMD/BMI) ratio, and (**C**) total hip bone mineral density/body mass index (TH BMD/BMI) ratio in the control group (C), and obese women evaluated one year (B1) and five (B5) years after Roux-en-Y gastric bypass. Data are reported as means±SD. *P<0.05, ANOVA.

Lumbar spine BMD was higher in the B1 group (1.125±0.11 g/cm^2^) compared with the C (0.990±0.11 g/cm^2^) and B5 groups (1.016±0.15 g/cm^2^, P=0.006). Femoral neck BMD was higher in the B1 group (0.929±0.12 g/cm^2^) compared with the C group (0.808±0.096 g/cm^2^, P=0.01), but showed no significant differences between the two bariatric groups (B5=0.849±0.13 g/cm^2^). Total hip BMD was similar across the three groups (C=0.916±0.11 g/cm^2^, B1=1.005±0.13 g/cm^2^, B5=0.931±0.18 g/cm^2^, P=0.13). In contrast, the BMD/BMI ratios in the lumbar spine and total hip were higher in the C group compared with the other two groups, while the ratio in the femoral neck was higher in the C group compared with the B5 group (Figure 1, Table 2). All subjects in the C and B1 groups had BMD Z-scores above -2.0, while two individuals in the B5 group had a low age-adjusted bone mass in the lumbar spine.

**Table 2:** Dual-energy X-ray absorptiometry and magnetic resonance results of the control group, and obese women evaluated one year (B1) and five (B5) years after Roux-en-Y gastric bypass.

	Control group (n=21)	B1 group (n=16)	B5 group (n=17)
Total hip BMD/BMI (g/cm^2^/kg/m^2^)	0.040±0.007^a^	0.033±0.006	0.029±0.007
Femoral neck BMD/BMI (g/cm^2^/kg/m^2^)	0.035±0.006^b^	0.030±0.007	0.026±0.006
L1-L4 BMD (g/cm^2^)/BMI (g/cm^2^/kg/m^2^)	0.043±0.006^a^	0.037±0.007	0.031±0.007
Subcutaneous adipose tissue (SAT) (mm^2^)	20910±8283^a^	37340±12231	42520±11653
Visceral adipose tissue (VAT) (mm^2^)	3308±2609	3760±2714	3544±2393
VAT/SAT	0.14±0.08^b^	0.10±0.06	0.08±0.05
Intrahepatic lipids (IHL) (%)	1.9±1.8	1.6±0.9	3.0±2.8

Data are reported as means±SD. ^a^P<0.05 compared to B1 and B5, ^b^P<0.05 compared to B5 (ANOVA).

**Figure 2:**
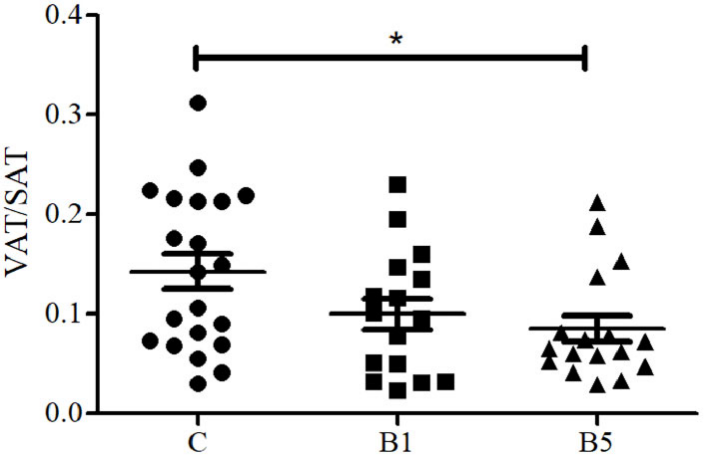
Visceral adipose tissue (mm^2^)/subcutaneous adipose tissue (mm^2^) (VAT/SAT) ratio in the control group (C), and obese women evaluated one year (B1) and five (B5) years after Roux-en-Y gastric bypass. Measurements were obtained by magnetic resonance imaging. Data are reported as means±SD. *P<0.05, ANOVA.

The SAT values were significantly greater in the B1 and B5 groups compared with the C group. These differences were also observed in the SAT/BMI ratios, while VAT values were similar across the three groups. The VAT/SAT ratio was greater in the C group compared with the B5 group, while no differences were observed between the B1 and the other two groups (Figure 2). No significant differences were observed regarding the amounts of IHL among the groups.

## Discussion

Technology has helped shift the focus of disease management from traumatic invasive procedures to less invasive and more functional therapies. However, the treatment of severe obesity has followed the opposite direction. After frustrating experiences with the clinical treatment of obesity, bariatric surgery has emerged as the most effective therapy for rapid and long-lasting weight loss.

The present study is aligned with previously published data showing metabolic benefits from weight loss after RYGB (5). One and five years after the procedure, the patients exhibited normal serum insulin levels and HOMA-IR values despite continuing to be overweight or obese. The present study contributes to existing data by showing that patients submitted to RYGB exhibit a healthy distribution of white adipose tissue (WAT). The B1 and B5 groups presented greater SAT than the control group, a pattern of adipose tissue distribution with metabolic advantages according to several studies. Although the mean BMI levels in both bariatric groups remained high, deposition of fat in the liver was not increased in these groups. On the other hand, this study showed that RYGB was associated with long-term bone catabolism, detected by persistently elevated serum CTX levels without increased osteocalcin. While individuals in the B1 group had BMD levels greater than those in the C group, the same difference was not observed between individuals in the B5 and C groups. Our study also showed that the BMD/BMI ratio tends to decline over time.

The efficacy of RYGB in treating severe obesity has been recognized in several studies, including studies showing reduced mortality in patients undergoing RYGB compared with matched controls not submitted to this procedure (27,28). Substantial changes in fat content and, particularly, fat distribution after RYGB have been observed in previous studies describing a decrease in hepatic, pancreatic, and cardiac fat (27,29). In line with these studies, the present investigation suggested that RYGB enabled individuals to acquire an early, persistent, and healthy distribution of adipose tissue, reflected by increased SAT and similar VAT, when patients submitted to RYGB are compared with controls (30).

The definition of healthy body fat distribution has been based on the distribution of adipose tissue in metabolically healthy individuals with obesity and metabolically unhealthy individuals with normal weight. The former has higher SAT values, while the latter has higher VAT values. A healthy fat distribution not only prevents insulin resistance but also allows the retention of lipids within proper locations of fat storage, preventing lipid overflow from the adipose tissue to other sites (30). In a recent study from our group, we described a spectrum of VAT and IHL variations. Both IHL and VAT were increased in individuals with obesity and T2DM compared with those who were obese but had no diabetes, who in turn had greater IHL and VAT values than lean individuals with normal glucose levels (25). A limitation in the capacity of adipocytes to store lipids and the increased lipolytic activity of VAT compared with SAT are determinants of lipid spillover from WAT into other tissues, especially in central obesity (31). The lipid spillover combined with the inflammatory profile of endocrine/paracrine/autocrine secretion of engorged adipocytes creates a toxic environment that culminates in insulin resistance, T2DM, steatohepatitis, and cardiovascular diseases (32). RYGB seems to shut off factors that are important in maintaining the vicious cycle that emerges during weight gain. The results of the present study suggest preferential shrinkage of VAT and maintenance of SAT in individuals with early and enduring weight loss after RYGB. We can hypothesize that the preferential storage of fat within the SAT after RYGB is important in preventing insulin resistance, T2DM, and steatohepatitis. The observed normal HOMA-IR values and serum glucose, insulin, ALT, and AST levels after RYGB reflect an improved metabolic status. The metabolic environment in patients submitted to RYGB contrasts with that observed in individuals with short bowel syndrome, who are prone to present persistent hepatic steatosis after withdrawal of parenteral nutrition (23).

The adipokines leptin and adiponectin are intricately linked to the amount of adipose tissue, but their levels fluctuate in opposite directions, i.e., leptin increases during weight gain, whereas adiponectin - an anti-inflammatory and insulin-sensitizing adipokine - increases with weight loss. Aligned with previous evidence (33,34), the findings of the present study showed that individuals undergoing weight loss after RYGB exhibited normal leptin and adiponectin profiles despite not attaining normal weight (35). Metabolic improvement before substantial weight loss most likely reflects increased intestinal secretion of GLP1 (36). Although the mechanisms involved in bone loss after RYGB remain unclear, they may involve a combination of factors including secondary hyperparathyroidism and decreased levels of insulin and IGF1. In line with this hypothesis, the B5 group in the present study exhibited significantly higher mean levels of serum PTH than the C and B1 groups but lower mean levels of serum IGF1 than the C group (37). There is a general recognition that obesity is associated with decreased serum 25-OHD levels, supposedly due to sequestration of cholecalciferol by the adipose tissue (38). However, conflicting data have been reported in the literature concerning 25-OHD sufficiency after RYGB. For instance, a retrospective study by Johnson et al. (39) reported a prevalence of vitamin D deficiency of 11% during the first year after RYGB, which increased to 20.3% thereafter. During rapid weight loss, the abrupt shrinkage of adipose tissue has been hypothesized to release stored vitamin D into circulation (17). Aligned with this hypothesis, the findings of the present study suggested that secondary hyperparathyroidism may not be an early occurrence after RYGB, but an additional component in the complex process compromising bone homeostasis during body weight recalibration after the procedure.

It is well known that body weight has a positive effect on bone mass, as reflected by greater bone mass in obese individuals compared with normal weight controls (15). As such, short-term and even long-term cross-sectional measurements of BMD may not detect abnormal bone mass after RYGB. On the other hand, the present findings reveal two aspects of the abnormal bone status after RYGB: first, increased circulating CTX levels one and five years after the procedure, and second, a lower BMD at all sites (lumbar spine, femoral neck, and total hip) five years after RYGB (B5 group compared with controls), as reflected by the BMD/BMI ratio. Accordingly, previous studies have shown that after approximately one year, BMD levels in individuals submitted to RYGB are still above those in controls (17), and that the sustained increase in CTX levels is maintained for at least five years (40).

Limitations of the present study include the cross-sectional design and the small sample size. However, there is sufficient physiopathologic evidence supporting the findings reported here. The strengths of the study are the exclusive enrollment of women and the comparable levels of body weight and BMI among the patients in both bariatric groups.

In conclusion, RYGB offers a perspective for long-term weight loss but is also associated with adverse effects. The present study supports the existing evidence showing long-term positive effects of RYGB on insulin resistance and reduced fat accumulation in the liver. These results call attention to the lasting effects of bone catabolism after bariatric surgery, as reflected by elevated serum levels of CTX and secondary hyperparathyroidism. BMD deterioration may be masked by an elevated BMD prior to RYGB, but defective bone mass adjustment is observed in a lower BMD/BMI ratio five years after the surgery.
